# 3-(Phenyl­carbamoyl)acrylic acid

**DOI:** 10.1107/S1600536812035581

**Published:** 2012-08-23

**Authors:** Shouwen Jin, Yanfei Huang, Shuaishuai Wei, Yong Zhou, Yingping Zhou

**Affiliations:** a Tianmu College of ZheJiang A & F University, Lin’An 311300, People’s Republic of China

## Abstract

In the title compound, C_10_H_9_NO_3_, the dihedral angle between the phenyl ring and the amide group is 10.8 (2)°. The C=O and O—H bonds of the carboxyl group adopt an *anti* orientation and an intra­molecular O—H⋯O hydrogen bond closes an *S*(7) ring. In the crystal, N—H⋯O hydrogen bonds link the mol­ecules into *C*(7) chains propagating in [101]. The packing is consolidated by C—H⋯O inter­actions, generating sheets aligned at an angle of *ca* 60° with the *bc* plane.

## Related literature

For background to carb­oxy­lic acids in supra­molecular chemistry, see: Grossel *et al.* (2006 ▶). For a related structure, see: Jin *et al.* (2010 ▶).
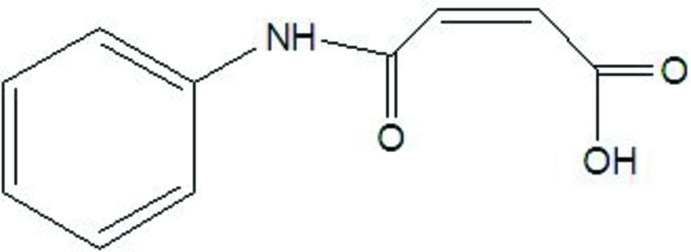


## Experimental

### 

#### Crystal data

C_10_H_9_NO_3_
*M* *_r_* = 191.18
Monoclinic, 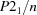

*a* = 7.2396 (8) Å
*b* = 10.5918 (11) Å
*c* = 11.7718 (15) Å
β = 99.122 (1)°
*V* = 891.25 (18) Å^3^
*Z* = 4
Mo *K*α radiation
μ = 0.11 mm^−1^
*T* = 298 K
0.38 × 0.36 × 0.33 mm

#### Data collection

Bruker SMART CCD diffractometer
Absorption correction: multi-scan (*SADABS*; Bruker, 2002 ▶) *T*_min_ = 0.960, *T*_max_ = 0.965
5497 measured reflections
2190 independent reflections
1356 reflections with *I* > 2σ(*I*)
*R*_int_ = 0.043

#### Refinement

*R*[*F* ^2^ > 2σ(*F* ^2^)] = 0.049
*wR*(*F* ^2^) = 0.157
*S* = 1.03
2190 reflections
128 parameters
H-atom parameters constrained
Δρ_max_ = 0.20 e Å^−3^
Δρ_min_ = −0.26 e Å^−3^

### 

Data collection: *SMART* (Bruker, 2002 ▶); cell refinement: *SAINT* (Bruker, 2002 ▶); data reduction: *SAINT*; program(s) used to solve structure: *SHELXS97* (Sheldrick, 2008 ▶); program(s) used to refine structure: *SHELXL97* (Sheldrick, 2008 ▶); molecular graphics: *SHELXTL* (Sheldrick, 2008 ▶); software used to prepare material for publication: *SHELXTL*.

## Supplementary Material

Crystal structure: contains datablock(s) global, I. DOI: 10.1107/S1600536812035581/hb6935sup1.cif

Structure factors: contains datablock(s) I. DOI: 10.1107/S1600536812035581/hb6935Isup2.hkl

Supplementary material file. DOI: 10.1107/S1600536812035581/hb6935Isup3.cml

Additional supplementary materials:  crystallographic information; 3D view; checkCIF report

## Figures and Tables

**Table 1:** Hydrogen-bond geometry (Å, °)

*D*—H⋯*A*	*D*—H	H⋯*A*	*D*⋯*A*	*D*—H⋯*A*
O1—H1⋯O3	0.82	1.68	2.4947 (19)	175
N1—H1*A*⋯O2^i^	0.86	2.04	2.885 (2)	169
C9—H9⋯O3^ii^	0.93	2.51	3.389 (2)	157
C10—H10⋯O2^i^	0.93	2.58	3.335 (2)	138

Symmetry codes: (i) 
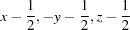
; (ii) 
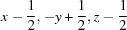
.

**Fig. 1.**
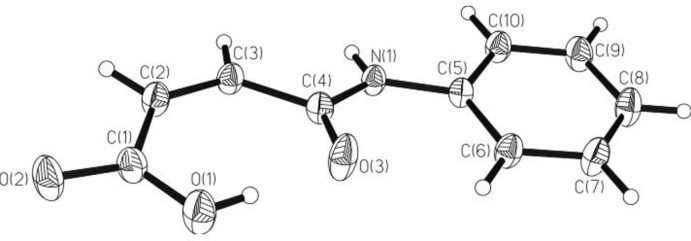
The structure of the title compound, showing displacement ellipsoids drawn at the 30% probability level.

**Fig. 2.**
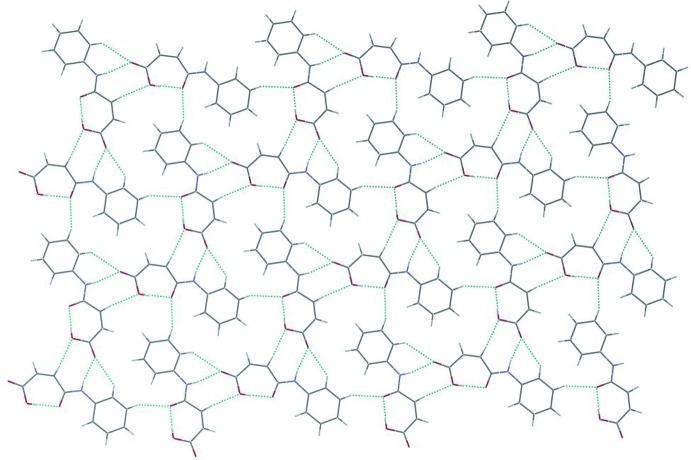
Two-dimensional sheet structure formed through hydrogen bonds.

## supplementary crystallographic information

### Comment

The carboxylic acid contains the important hydrogen bonding functional group for crystal engineering (Grossel *et al.*, 2006). As an extension of our study concentrating on hydrogen bonded assembly of organic acid and organic base (Jin *et al.*, 2010), herein we report the crystal structure of 3-phenylcarbamoyl-acrylic acid.

The single-crystal of the title compound (Fig.1) with the formula C_10_H_9_NO_3_ was obtained by recrystallization of 3-phenylcarbamoyl-acrylic acid and 2-methylquinoline from a methanol solution. However the 2-methylquinoline molecules do not appear in the title compound. X-ray diffraction analysis indicated that the asymmetric unit of the structure contains one molecule. The conformations of the amide oxygen and the carbonyl oxygen of the acid segments are anti to each other and the amide oxygen is anti to the H atom on the olefinic group, while the carbonyl oxygen of the acid is *syn* to the CH at the olefinic group. Thus there existed intramolecular O—H···O hydrogen bond producing a S_1_^1^(7) ring.

The dihedral angle between the phenyl ring and the amide group in the molecule is 10.8 (2)°.

Two adjacent 3-phenylcarbamoyl-acrylic acids were joined together *via* the N—H···O, and CH—O interactions to form a dimer. Both carboxylic acids in the dimer were almost perpendicular with each other. In the dimer there are hydrogen-bonded ring motifs with descriptors of *R*_2_^1^(6), and *R*_2_^2^(8). The two ring motifs were fused together by the N—H···O hydrogen bond. The neighboring carboxylic dimers were linked together through the CH—O associations between the benzene CH and the carbonyl group with C—O distance of 3.389 Å to form one-dimensional chain. The one-dimensional chains were combined together by the interchain CH—O, and N—H···O interactions to form two-dimensional sheet extending at the direction that made a dihedral angle of *ca* 60° with the *bc* plane (Fig. 2). Two two-dimensional sheets were further held together by the intersheet C–π interactions with C···C_g_ distance of 3.369 Å to generate two-dimensional double sheet structure.

### Experimental

Crystals of 3-phenylcarbamoyl-acrylic acid were formed by slow evaporation of its methanol solution at room temperature. 3-phenylcarbamoyl-acrylic acid (19.1 mg, 0.10 mmol) was dissolved in 4 ml of methanol, and 2-methylquinoline (14.3 mg, 0.1 mmol) was added to the methanol solution. The solution was then filtered into a test tube and left standing at room temperature. After about one week colorless blocks crystals were obtained.

### Refinement

H atoms bonded to O, and N atoms were located in a difference Fourier map and refined isotropically.

Other H atoms were positioned geometrically with C—H = 0.93 Å for aromatic, and constrained to ride on their parent atoms with *U*_iso_(H) = 1.2Ueq(C).

### Crystal data

**Table:**

C_10_H_9_NO_3_	*Z* = 4
*M**_r_* = 191.18	*F*(000) = 400
Monoclinic, *P*2_1_/*n*	*D*_x_ = 1.425 Mg m^−^^3^
*a* = 7.2396 (8) Å	Mo *K*α radiation, λ = 0.71073 Å
*b* = 10.5918 (11) Å	µ = 0.11 mm^−^^1^
*c* = 11.7718 (15) Å	*T* = 298 K
β = 99.122 (1)°	BLOCK, colorless
*V* = 891.25 (18) Å^3^	0.38 × 0.36 × 0.33 mm

### Data collection

**Table:**

Bruker SMART CCD diffractometer	2190 independent reflections
Radiation source: fine-focus sealed tube	1356 reflections with *I* > 2σ(*I*)
Graphite monochromator	*R*_int_ = 0.043
phi and ω scans	θ_max_ = 28.3°, θ_min_ = 2.6°
Absorption correction: multi-scan (*SADABS*; Bruker, 2002)	*h* = −9→9
*T*_min_ = 0.960, *T*_max_ = 0.965	*k* = −14→8
5497 measured reflections	*l* = −15→14

### Refinement

**Table:**

Refinement on *F*^2^	Secondary atom site location: difference Fourier map
Least-squares matrix: full	Hydrogen site location: inferred from neighbouring sites
*R*[*F*^2^ > 2σ(*F*^2^)] = 0.049	H-atom parameters constrained
*wR*(*F*^2^) = 0.157	*w* = 1/[σ^2^(*F*_o_^2^) + (0.0804*P*)^2^ + 0.0617*P*] where *P* = (*F*_o_^2^ + 2*F*_c_^2^)/3
*S* = 1.03	(Δ/σ)_max_ < 0.001
2190 reflections	Δρ_max_ = 0.20 e Å^−^^3^
128 parameters	Δρ_min_ = −0.26 e Å^−^^3^
0 restraints	Extinction correction: *SHELXL*, Fc^*^=kFc[1+0.001xFc^2^λ^3^/sin(2θ)]^-1/4^
Primary atom site location: structure-invariant direct methods	Extinction coefficient: 0.020 (5)

### Special details

**Table:**

Geometry. All e.s.d.'s (except the e.s.d. in the dihedral angle between two l.s. planes) are estimated using the full covariance matrix. The cell e.s.d.'s are taken into account individually in the estimation of e.s.d.'s in distances, angles and torsion angles; correlations between e.s.d.'s in cell parameters are only used when they are defined by crystal symmetry. An approximate (isotropic) treatment of cell e.s.d.'s is used for estimating e.s.d.'s involving l.s. planes.
Refinement. Refinement of *F*^2^ against ALL reflections. The weighted *R*-factor *wR* and goodness of fit *S* are based on *F*^2^, conventional *R*-factors *R* are based on *F*, with *F* set to zero for negative *F*^2^. The threshold expression of *F*^2^ > σ(*F*^2^) is used only for calculating *R*-factors(gt) *etc*. and is not relevant to the choice of reflections for refinement. *R*-factors based on *F*^2^ are statistically about twice as large as those based on *F*, and *R*- factors based on ALL data will be even larger.

### Fractional atomic coordinates and isotropic or equivalent isotropic displacement parameters (Å^2^)

**Table:**

	*x*	*y*	*z*	*U*_iso_*/*U*_eq_	
O1	1.0940 (2)	−0.19833 (13)	0.83751 (12)	0.0561 (5)	
H1	1.0527	−0.1373	0.7987	0.084*	
N1	0.7880 (2)	0.00622 (13)	0.53533 (12)	0.0340 (4)	
H1A	0.7299	−0.0337	0.4767	0.041*	
O2	1.0652 (2)	−0.40422 (14)	0.83105 (12)	0.0546 (4)	
C4	0.8638 (3)	−0.06557 (17)	0.62392 (15)	0.0348 (4)	
O3	0.9581 (2)	−0.02077 (13)	0.71212 (13)	0.0585 (5)	
C3	0.8248 (3)	−0.20179 (16)	0.60914 (15)	0.0358 (4)	
H3	0.7460	−0.2246	0.5421	0.043*	
C5	0.7910 (2)	0.14022 (16)	0.52529 (14)	0.0326 (4)	
C10	0.6725 (3)	0.19311 (17)	0.43353 (16)	0.0401 (5)	
H10	0.5983	0.1413	0.3811	0.048*	
C6	0.9055 (3)	0.21772 (18)	0.60146 (16)	0.0414 (5)	
H6	0.9883	0.1831	0.6619	0.050*	
C2	0.8880 (3)	−0.29664 (17)	0.67949 (16)	0.0395 (5)	
H2	0.8378	−0.3748	0.6554	0.047*	
C1	1.0230 (3)	−0.30190 (18)	0.78871 (16)	0.0395 (5)	
C9	0.6645 (3)	0.32210 (18)	0.41997 (17)	0.0476 (5)	
H9	0.5842	0.3573	0.3586	0.057*	
C7	0.8946 (3)	0.34771 (19)	0.58618 (17)	0.0487 (5)	
H7	0.9698	0.4001	0.6374	0.058*	
C8	0.7752 (3)	0.39960 (19)	0.49705 (18)	0.0496 (6)	
H8	0.7683	0.4868	0.4882	0.060*	

### Atomic displacement parameters (Å^2^)

**Table:**

	*U*^11^	*U*^22^	*U*^33^	*U*^12^	*U*^13^	*U*^23^
O1	0.0747 (11)	0.0394 (9)	0.0446 (9)	0.0003 (7)	−0.0197 (7)	0.0041 (6)
N1	0.0425 (9)	0.0265 (8)	0.0302 (8)	−0.0006 (6)	−0.0026 (6)	−0.0013 (6)
O2	0.0712 (11)	0.0410 (9)	0.0462 (9)	0.0107 (7)	−0.0073 (7)	0.0127 (7)
C4	0.0423 (10)	0.0309 (10)	0.0290 (9)	0.0009 (8)	−0.0007 (7)	−0.0001 (7)
O3	0.0913 (12)	0.0311 (8)	0.0420 (8)	−0.0062 (7)	−0.0235 (7)	0.0000 (6)
C3	0.0426 (10)	0.0298 (10)	0.0324 (9)	−0.0020 (8)	−0.0023 (7)	−0.0014 (7)
C5	0.0402 (10)	0.0272 (9)	0.0298 (9)	0.0003 (7)	0.0043 (7)	0.0001 (7)
C10	0.0482 (11)	0.0320 (10)	0.0367 (10)	−0.0003 (8)	−0.0038 (8)	0.0016 (8)
C6	0.0533 (12)	0.0332 (11)	0.0346 (10)	−0.0037 (8)	−0.0022 (8)	0.0009 (8)
C2	0.0492 (12)	0.0294 (10)	0.0375 (10)	−0.0016 (8)	−0.0006 (8)	−0.0006 (8)
C1	0.0468 (12)	0.0357 (11)	0.0347 (10)	0.0038 (8)	0.0022 (8)	0.0047 (8)
C9	0.0596 (13)	0.0371 (11)	0.0414 (11)	0.0067 (10)	−0.0061 (9)	0.0071 (9)
C7	0.0679 (15)	0.0329 (11)	0.0420 (11)	−0.0073 (10)	−0.0018 (10)	−0.0023 (9)
C8	0.0715 (15)	0.0297 (11)	0.0459 (12)	0.0000 (10)	0.0041 (11)	0.0037 (9)

### Geometric parameters (Å, º)

**Table:**

O1—C1	1.305 (2)	C10—C9	1.376 (2)
O1—H1	0.8200	C10—H10	0.9300
N1—C4	1.336 (2)	C6—C7	1.389 (3)
N1—C5	1.425 (2)	C6—H6	0.9300
N1—H1A	0.8600	C2—C1	1.488 (3)
O2—C1	1.212 (2)	C2—H2	0.9300
C4—O3	1.243 (2)	C9—C8	1.382 (3)
C4—C3	1.475 (2)	C9—H9	0.9300
C3—C2	1.336 (3)	C7—C8	1.365 (3)
C3—H3	0.9300	C7—H7	0.9300
C5—C10	1.387 (3)	C8—H8	0.9300
C5—C6	1.389 (3)		
			
C1—O1—H1	109.5	C5—C6—H6	120.4
C4—N1—C5	128.47 (15)	C7—C6—H6	120.4
C4—N1—H1A	115.8	C3—C2—C1	132.65 (18)
C5—N1—H1A	115.8	C3—C2—H2	113.7
O3—C4—N1	122.54 (17)	C1—C2—H2	113.7
O3—C4—C3	122.77 (17)	O2—C1—O1	120.98 (18)
N1—C4—C3	114.68 (15)	O2—C1—C2	118.50 (18)
C2—C3—C4	128.48 (17)	O1—C1—C2	120.51 (16)
C2—C3—H3	115.8	C10—C9—C8	120.30 (19)
C4—C3—H3	115.8	C10—C9—H9	119.9
C10—C5—C6	119.76 (17)	C8—C9—H9	119.9
C10—C5—N1	116.85 (16)	C8—C7—C6	120.97 (19)
C6—C5—N1	123.40 (16)	C8—C7—H7	119.5
C9—C10—C5	120.07 (18)	C6—C7—H7	119.5
C9—C10—H10	120.0	C7—C8—C9	119.76 (19)
C5—C10—H10	120.0	C7—C8—H8	120.1
C5—C6—C7	119.11 (18)	C9—C8—H8	120.1
			
C5—N1—C4—O3	−3.0 (3)	N1—C5—C6—C7	−178.30 (17)
C5—N1—C4—C3	176.37 (16)	C4—C3—C2—C1	−4.5 (4)
O3—C4—C3—C2	−4.1 (3)	C3—C2—C1—O2	−174.8 (2)
N1—C4—C3—C2	176.5 (2)	C3—C2—C1—O1	5.2 (3)
C4—N1—C5—C10	−168.15 (18)	C5—C10—C9—C8	0.4 (3)
C4—N1—C5—C6	12.2 (3)	C5—C6—C7—C8	−0.8 (3)
C6—C5—C10—C9	−1.9 (3)	C6—C7—C8—C9	−0.7 (3)
N1—C5—C10—C9	178.43 (17)	C10—C9—C8—C7	0.8 (3)
C10—C5—C6—C7	2.0 (3)		

### Hydrogen-bond geometry (Å, º)

**Table:**

*D*—H···*A*	*D*—H	H···*A*	*D*···*A*	*D*—H···*A*
O1—H1···O3	0.82	1.68	2.4947 (19)	175
N1—H1*A*···O2^i^	0.86	2.04	2.885 (2)	169
C9—H9···O3^ii^	0.93	2.51	3.389 (2)	157
C10—H10···O2^i^	0.93	2.58	3.335 (2)	138

Symmetry codes: (i) *x*−1/2, −*y*−1/2, *z*−1/2; (ii) *x*−1/2, −*y*+1/2, *z*−1/2.
